# Facile fabrication of HDPE-g-MA/nanodiamond nanocomposites via one-step reactive blending

**DOI:** 10.1186/1556-276X-7-355

**Published:** 2012-06-29

**Authors:** Ping’an Song, Youming Yu, Qiang Wu, Shenyuan Fu

**Affiliations:** 1 Department of Materials, College of Engineering, Zhejiang Agriculture & Forestry University, Lin’an, Hangzhou, 311300, China; 2 Institute of Polymer Composites, Zhejiang University, Hangzhou, 310027, China

**Keywords:** Nanodiamond, Polyethylene, Nanocomposites, Flammability, Mechanics

## Abstract

In this letter, nanocomposites based on maleic anhydride grafted high density polyethylene (HDPE-g-MA) and amine-functionalized nanodiamond (ND) were fabricated via one-step reactive melt-blending, generating a homogeneous dispersion of ND, as evidenced by transmission electron microscope observations. Thermal analysis results suggest that addition of ND does not affect significantly thermal stability of polymer matrix in nitrogen. However, it was interestingly found that incorporating pure ND decreases the thermal oxidation degradation stability temperature, but blending amino-functionalized ND via reactive processing significantly enhances it of HDPE in air condition. Most importantly, cone tests revealed that both ND additives and reactive blending greatly reduce the heat release rate of HDPE. The results suggest that ND has a potential application as flame retardant alternative for polymers. Tensile results show that adding ND considerably enhances Young’s modulus, and reactive blending leads to further improvement in Young’s modulus while hardly reducing the elongation at break of HDPE.

## Background

With the increasingly rapid need for synthetic polymers due to their various advantages over conventional materials, their inherent flammability, however, is gradually limiting their many potential applications for safety considerations [1]. Thus, it is rather crucial to explore nontoxic and environmentally friendly flame retardancy approaches for polymer materials [2]. Among various polymers, polyolefin is much more flammable due to its saturated hydrocarbon structure and non-char-forming nature. Recently, nanocomposites have been reported to significantly reduce the flammability properties of polymers only by adding very low loading of nanoscale additives, offering an alternative to conventional flame retardants [1,3,4].

So far, layered silicates or clay [5-7], carbon-based nanomaterials like carbon nanotubes (CNTs) [1,8-10], C_60_[11-13], and polyhedral oligomeric silsesquioxanes [14,15] have been reported to improve significantly the thermal stability and flame retardancy of polymers. Recently, another carbon-based nanomaterial, nanodiamond (ND), has been widely used in the field of material science and engineering because of its unique properties such as the highest bulk modulus, high wear resistance, superior thermal conductivity and stability, excellent electrical insulating, and outstanding tribological properties[16,17]. It has been reported that the addition of ND could improve the mechanical and thermal properties of polymeric materials such as epoxy resin [18], poly(vinyl alcohol) [17,19], and poly(lactic acid) [20]. Recently, Behler et al. [21] prepared composites nanofibers with high loading of ND via electrospun method which exhibited significantly enhanced tensile strength and Young’s modulus. Since CNTs and C_60_ can enhance the thermal stability and flame retardancy of polymer materials, thus ND, as their homogeneous carbon material, should also exhibit this capability. However, ND has not been employed as a potential flame retardant for polymeric materials like flammable polyolefins to date.

This communication will examine the effects of ND on thermal stability, flammability, and mechanical performances of polymers, especially for polyolefin. In addition, various spectroscopic techniques have found that the surface of ND have various functional groups, mostly oxygenated moieties such as -COOH (carboxylic acid), lactone, C = O (keto carbonyl), -C-O-C (ether), -OH (hydroxyl), and as such [17,22]. Thus, to achieve a good dispersion of ND in polyolefin matrix, ND was modified by 3-aminopropyltriethyoxyl silane (APTES) to produce amine-functionalized nanodiamond (NH_2_-ND), and anhydride-grafted high density polyethylene (HDPE-g-MA) was chosen as the polymer matrix. By this way, we will create polymer nanocomposites with homogeneous dispersion of ND just by one-step reactive melt-blending strategy.

## Methods

### Materials

ND powder was purchased from Heyuan Zhonglian Nano-Tech, Co., Ltd., Heyuan City, Guangdong Province, China and produced by detonation of explosive compounds, including 2-methyl 1,3,5-trinitrobenzene and hexahydro-1,3,5-trinitro-1,3,5-triazine, under an inert atmosphere. Maleic HDPE-g-MA(CMG 9804) was purchased from Shanghai Kumho Sunny New Technology Development Co., Ltd., Minhang District, Shanghai, China with a maleic anhydride content of 0.9 wt.%. All chemical reagents like APTES, absolute ethanol, and other materials are received and used without further purification.

### Fabrication of functionalized ND and its nanocomposites

Amine-functionalized nanodiamond (NH_2_-ND) was fabricated by the following procedure. Typically, 50 mg ND powder was dispersed in absolute ethanol via sonication for 30 min and then, an excess solution of APTES was slowly dropped and stirred at 80 °C for 8 h to complete the reaction. The NH_2_-ND was filtered and subsequently washed with ethanol for at least 5 times, which then was dried at 80 °C under vacuum for 12 h.

HDPE/ND nanocomposites were fabricated via a melt-blending method using a Thermo Haake mixer (Thermo Scientific, Boston, USA) with a rotation speed of 60 rpm at 180 °C for 15 min to enable the *in situ* reaction complete. Then, composites were compression-molded at 185 °C under 10 MPa into sheets with certain sizes and shapes for a particular test. HDPE composites with NH2-ND loading of 1.0 and 2.0 wt.% were designated as HPgND-1% and HPgND-2%, and HDPE/ND with 1.0 wt.% of ND was also prepared using the same protocol as a comparison named HPmND-1%.

### Characterization

Infrared spectrometry (IR, Vector-22 FT-IR, Bruker Corporation, Billerica, MA, USA) was used to characterize ND, NH_2_-ND, and the corresponding changes during reactive blending process. The morphology of ND, NH_2_-ND, and their dispersion were observed using transmission electron microscopy (TEM, JEM-1200EX, JEOL Ltd., Tokyo, Japan). Thermogravimetric analyses (TGA) was performed on a TA SDTQ600 thermal analyzer (TA Instruments, New Castle, DE, USA) at a scanning rate of 20 °C/min in air and N_2_ with a temperature range of 50 °C to approximately 800 °C. Cone calorimeter tests were performed using a dual cone calorimeter (Fire Testing Technology (FTT), London, UK) according to ISO 5660 at an incident flux of 35 kWm^−2^, and the size of specimens was 100 × 100 × 3.0 mm; each sample was performed in triplicate. The data reported herein from cone calorimetry are reproducible to within around ±10 %. Rheological properties were evaluated using an advanced rheological expanded system with parallel plate geometry of 25 mm in diameter.

## Results and discussion

### Morphology

**Figure 1:**
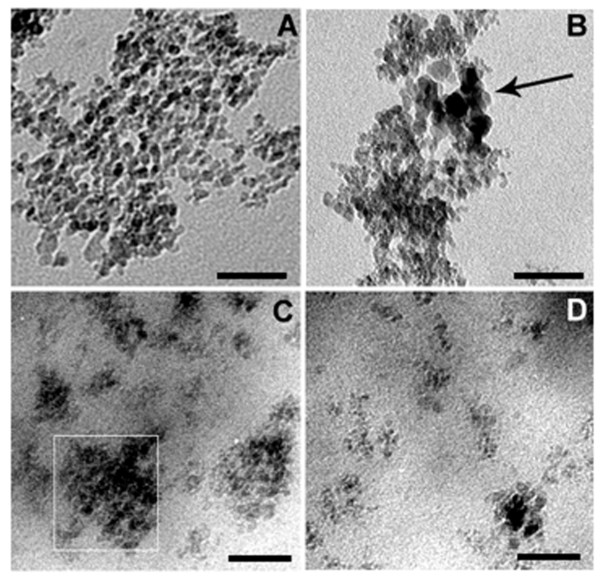
**TEM images of pristine.** (**A**) ND, (**B**) NH_2_-ND, (**C**) HPmND-1.0 %, and (**D**) HPgND-1.0 %. The scale bar is 50 nm.

As shown in Figure 1, unmodified ND powder displays big agglomerate clusters with a size of approximately 10 nm for individual ND particles (Figure 1A). In comparison, NH2-ND exhibits a relative loose agglomerates structure accompanied by slight size of ND particles (approximately 15 nm), indicating an effective surface modification (Figure 1B). For their corresponding nanocomposites, the majority of ND fillers still aggregate with different size of agglomerates (marked by square in Figure 1C), indicating a poor dispersion for HPmND-1%. While for HPgND-1%, uniform dispersion of ND particles can be readily observed only with some small and loose clusters, which demonstrates that one-step reactive blending was effective for improving the dispersion of ND in the polymer matrix.

### Infrared spectroscopy

**Figure 2:**
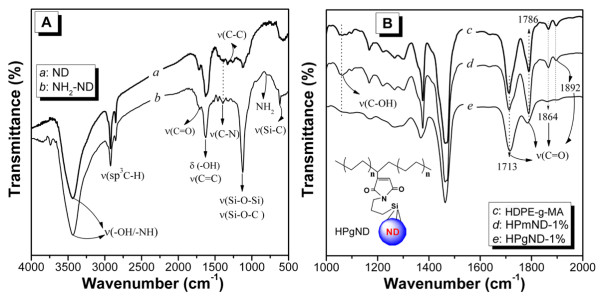
**FT-IR spectra of pristine composites.** (**A**) a, ND; b, NH_2_-ND; (**B**) c, HDPE-g-MA; d, HPmND-1.0%; and e, HPgND-1.0%.

To further characterize the structure of NH_2_-ND and the reactive blending process, infrared spectra were obtained, as shown in Figure 2. Besides the absorption peaks at 2,830 to approximately 2,960 cm^−1^ (υC-H) and 1,300 to 1,400 cm^−1^ (deformation vibration of alkyl groups), pure ND exhibits many other characteristic peaks [23]. The band at 1,640 cm^−1^ can be assigned to the stretching vibration of aromatic sp^2^ carbon bond (υC = C) related to graphite structure [17]. Another band at 1,733 cm^−1^ belongs to υC = O of carbonyl or carboxyl groups at around 1,100 to approximately 1,200 cm^−1^ of the υC-O in ethers [17,24]. In the spectra area, strong absorption bands are attributed to the stretching vibration of hydroxyl groups around 3,300 to approximately 3,500 cm^−1^, accompanied by its bending vibration at 1,620 to 1,640 cm^−1^[1,17,24] which partially originated from the hydroxyl groups of adsorbed water on the surface of ND particles. After being modified by APTES, several remarkable changes take place. The absorption peaks at 630 and 1,120 cm^−1^ are attributed to the stretching vibrations of Si-C bond and Si-O-Si or Si-O-C bond [12]. In addition, new bands at 806 and 1,400 cm^−1^ may be assigned to stretching vibration of C-N and bending vibration of -NH_2_. These changes strongly indicated that ND was successfully amino-functionalized with APTES (see Figure 2A).

As shown in Figure 2B, for the host HDPE-g-MA, two strong absorption peaks located at 1,864 and 1,892 cm^−1^ are attributed to asymmetric stretching of anhydride carbonyl groups (O = C-O-C = O), whereas the major bands centered at 1,786 and 1,713 cm^−1^ correspond to the anhydride symmetric carbonyl groups and carboxylic groups stretching vibration, respectively in Figure 2B-c[25,26]. The spectrum of HPmND does not show considerable differences when compared to the host polymer matrix, but the relative intensity of bands at 1,786 and 1,713 cm^–1^ changes which are most likely due to the superposition effect of carbonyl groups and to carboxylic groups from ND and those from HDPE-g-MA. However, these observations strongly demonstrate that only small changes in the molecular structure of HDPE-g-MA are brought by blending with ND even if some hydroxyl groups are on its surface in Figure 2B-d. In the contrary, by comparing the spectra for HDPE-g-MA and HDPE-g-MA/NH_2_-ND, significant changes of the IR absorptions occur in the carbonyl region, implying that a reaction has occurred between the MA and -NH_2_ groups upon blending for 15 min. Actually, both peaks at 1,860 and 1,892 cm^−1^ almost disappear, and the peak at 1,786 cm^−1^ reduces strongly in intensity and shifts to lower wave numbers for HPgND-1%. In response the relative intensity of peak at 1,713 cm^−1^ increases dramatically in Figure 2B-e. These findings strongly suggest the occurrence of a reaction between MA and NH_2_ groups with the formation of imide groups, in good agreement with the references [25,27].

### Thermal behavior

**Figure 3:**
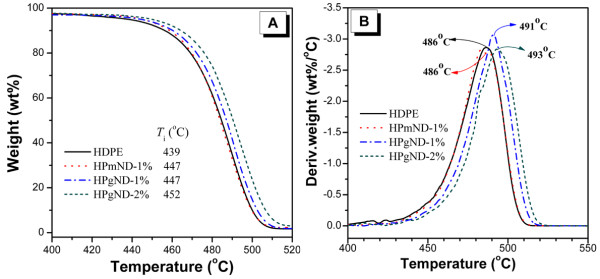
**TGA and DTG curves of weight loss rate.** (**A**) TGA and (**B**) DTG curves of weight loss rate by the initial sample mass at a heating rate of 20 °C/min in nitrogen condition. Notes: *T*_i_ and *T*_max_ were the temperatures where 5 wt.% weight loss and maximum weight loss rate occurred, respectively.

Normalized weight loss rate (or derivative weight) divided by the heating rate measured by TGA for the four samples in nitrogen is plotted in Figure 3. These results show that HDPE-g-MA starts to degrade at about 439 °C (*T*_i_), and degrades rapidly with a large single peak starting around 400 °C in nitrogen. This peak corresponds to the thermal degradation of polyethylene (PE) initiated primarily by thermal scissions of C-C chain bonds with a consequent formation of radical species [8,28]. The maximum weight loss temperature (*T*_max_) of polymer matrix takes place at ca.486 °C and completely degrades after 520 °C. Adding of 1% pristine ND seems not to result in significant changes, with a slightly delayed initial degradation temperature at ca. 447 °C, and the same broad single *T*_max_ as pure polymer matrix. With incorporation of 1% and 2% NH_2_-ND into HDPE, the *T*_i_ values were increased to 447 °C and 452 °C and 8 °C and 13 °C higher than that of HDPE (see Figure 3A). Moreover, the temperatures at the peak weight loss rates (*T*_max_) are about 5 °C and 7 °C higher than that of the polymer matrix (see Figure 3B), which was lower than those for PE/CNTs systems since the better barrier effects of nanotubes network [28]. Tang et al. [29] recently found that addition of 1% nanoscale carbon black (nano-CB) significantly enhanced the *T*_i_ by approximately 31 °C and *T*_max_ by approximately 7 °C in comparison with the polypropylene (PP) matrix. Li et al. [30] also observed that the adding of 2% nano-CaCO_3_ into epoxy resin hardly affected the *T*_i_ except for a slightly increased *T*_max_ compared to neat epoxy. In addition, the amount of ND addition does not affect the thermal stability of nanocomposites. However, the enhanced thermal stability of PE is mostly like due to the good dispersion, and the thermal protection of ND particles is due to its much higher thermal stability.

**Figure 4:**
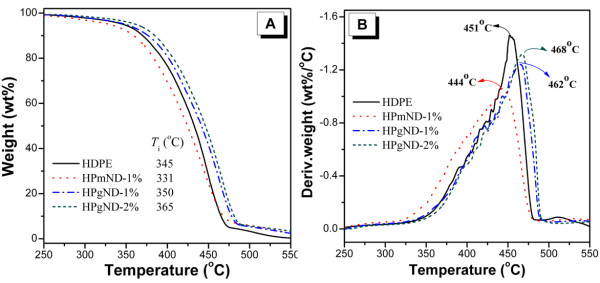
**TGA and DTG curves of normalized weight loss rate.** (**A**) TGA and (**B**) DTG curves of normalized weight loss rate by the initial sample mass at a heating rate of 20 °C/min in air condition. Notes: *T*_i_ and *T*_max_ were the temperatures where 5 wt.% weight loss and maximum weight loss rate occurred , respectively. *T*_max_ was obtained from the peak of weight loss in DTG curves.

Investigating the thermal degradation of samples in air is much more important than in nitrogen atmosphere, since most polymer materials are usually used in air. It was reported that PE in air is prominently decomposed by oxidative dehydrogenation accompanied by the formation of peroxy radicals that can decompose easily generating oxidized species [28]. As shown in Figure 4, pure HDPE-g-MA starts to degrade at about 345 *°*C (*T*_i_, defined as the temperature where 5 wt.% mass loss occurs) and decomposes most rapidly at about 451 *°*C without any char after ca.473 °C. Interestingly, addition of pristine ND leads to a slightly lower thermal oxidative stability relative to the polymer matrix. HPmND-1% initially degrades at about 331 *°*C, approximately 14 °C lower than that of pure HDPE-g-MA; the maximum mass rate temperature takes place at 444 °C, also approximately 7 °C lower than that of pure polymer. These results indicated a negative thermal stability effect of pristine ND on HDPE-g-MA, which is most likely attributed to the catalytic thermal oxidation degradation of oxygen-containing groups on surface of ND particles. Unlike pristine ND, HPgND-1% and HPgND-2% respectively exhibit initial degradation temperatures of 350 °C and 365 °C, and the latter is ca. 20 °C higher than that of pure polymer matrix, indicating an enhanced thermal stability of PE matrix. Moreover, their corresponding peak mass loss rate temperatures (*T*_max_) take place at ca. 462 °C and 468 °C, increased by 11 °C and 17 °C as compared to PE matrix, respectively. These values are also much lower than those of polyethylene/multiwalled carbon nanotubes (PE/MWNTs) systems [28], which is also due to the better thermal barrier properties of MWNTs network than ND particles of spherical shape. Compared to ND and MWNTs, addition of 1% nano-CB has increased *T*_i_ by approximately 17 °C and *T*_max_ by approximately 56 °C relative to neat PP in air condition due to the strong radical-scavenging action of carbon black [29]. However, ND can improve the thermal oxidative stability of PE to some extent. It should be noted that pristine ND reduces, while NH_2_-ND enhances the thermal oxidative stability of PE matrix; this interesting phenomenon is partially due to the improved dispersion of ND caused by the *in situ* reactive blending and partially because of catalytic degradation of oxygen-containing groups on the surface of ND particles.

### Flammability

**Figure 5:**
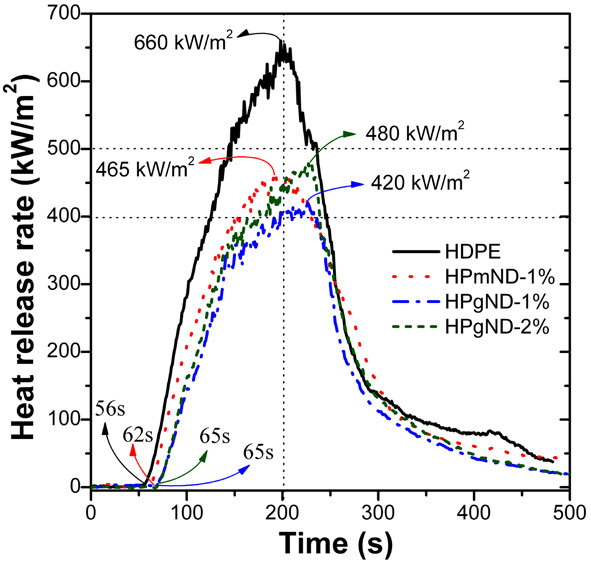
**Heat release rate curves of HDPE-g-MA and its nanocomposites at a heat flux of 35 kW/m**^**2**^**.**

Cone calorimeter measurements are widely used to evaluate the flammability of polymer materials [8,11-13]. As shown in Figure 5 and detailed data listed in Table 1, pure HDPE gives an time to ignition (*t*_ign_) of about 56 s, a peak heat release rate (PHRR) of ca. 660 kW/m^2^, and the time to PHRR which requires about 200 s at an incident heat flux of 35 kW/m^2^. After adding 1 wt.% pristine ND, the time to ignition is delayed to 62 s and the PHRR is reduced by 30% to 465 kW/m^2^. In comparison, incorporation of 1 wt.% NH_2_-ND leads to a time to ignition of 65 s, about 9 s longer than that of pure PE matrix. This prolonged time to ignition is rather significant because it can extend the ignition time to a fire and provide the time for people to find the possibility of a fire disaster. In addition, it reduced the PHRR by 36% (approximately 420 kW/m^2^) relative to pure PE, indicating a good flame retardancy. However, further increasing the amount of NH_2_-ND to 2 wt.%, the PHRR increases up to 480 kW/m^2^, demonstrating a reduction of flame retardancy even if maintaining at the same time, the ignition of 65 s. This trend is very similar to PP/MWNTs systems; 1.0 and 2.0 vol.% MWNTs result in a reduction of PHRR by approximately 73% and 68%, respectively. However, adding MWNTs into PP leads to a shorter time to ignition which is unfavorable for reducing the flammability of polymers [1,8]. Tang’s research [29] found that 1% nano-CB decreased the PHRR of PP by approximately 45% without reducing the time to ignition, and with increasing loading of nano-CB, the PHRR further reduced; but the *t*_ign_ was also shortened which was adverse to improving flame retardancy. The trends of both total heat release (THR) and average mass loss rate (AMLR) for all samples are basically similar to that of PHRR. Since ND cannot form three-dimensional network like CNTs [1] and such low loading level also cannot generate enough char residue to form a char layer; thus, the significant reduction in PHRR and THR suggests a considerable radical change in the combustion process of samples. Moreover, ND has a carbon of both sp^2^ and sp^3^ hybrids, which can, like C_60_, react with a free radical produced by the decomposition of a polymer during the combustion process [11].

**Table 1:** **Detailed data obtained from cone calorimeter measurements at an incident flux of 35 kW/m**^**2**^

**Sample ID**	***t***_**ign**_^**a**^**(s)**	**PHRR**^**a**^**(kW/m**^**2**^**)**	**THR**^**a**^**(MJ/m**^**2**^**)**	**AMLR**^**a**^**(g/s)**	**ASEA**^**a**^**(m**^**2**^**/kg)**
HDPE	56 ± 1	660 ± 20	84.8 ± 0.5	0.054 ± 0.03	414 ± 10
HPmND-1 %	62 ± 1	465 ± 15	79.5 ± 0.4	0.045 ± 0.02	320 ± 5
HPgND-1 %	65 ± 1	420 ±12	77.1 ± 0.3	0.043 ± 0.02	343 ± 7
HPgND-2 %	65 ± 1	480 ± 18	78.6 ± 0.5	0.049 ± 0.03	369 ± 8

Notes: ^a^*t*_ign_ means the time to ignition; AMLR, average mass loss rate; ASEA, average-specific extinction area; PHRR, peak of heat release rate; THR, total heat release.

Another important flame parameter, average specific extinction area (ASEA), should also be pointed out since it can evaluate the smoke-production capability during a fire and sometimes the smoke causes much more death than the heat in a real fire. The HDPE, HPmND-1%, HPgND-1%, and HPgND-2%, respectively, exhibit ASEA of 414, 320, 343, and 369 m^2^/kg, indicating that ND has certain capability of smoke suppression. Adding ND may cause the altering of the degradation and combustion mechanism of polymer and reduce the production of smoke particles. Whatever, this effect also contributes to improving the flame retardancy of polymer materials. As for the mechanism for both thermal degradation and flame retardancy of HDPE in the presence of ND, it is still being done presently and will be reported in the future.

### Tensile property

**Figure 6:**
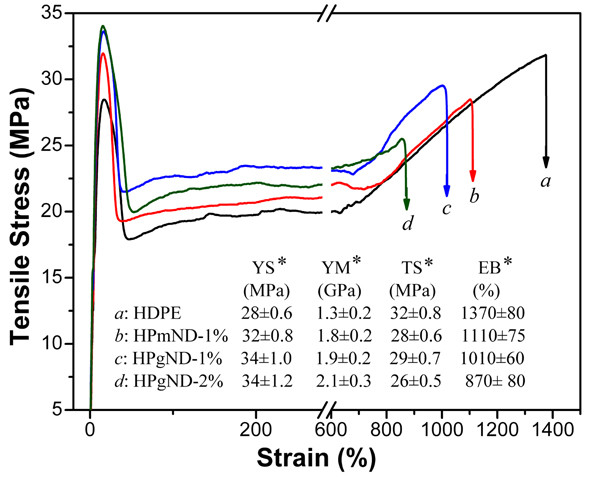
**Typical stress–strain curves of HDPE and its nanocomposites.** Notes: Asterisk in YS, YM, TS, and EB represents the yield stress, Young’s modulus, tensile stress, and elongation at break, respectively.

Typical stress–strain curves of HDPE and its nanocomposites are shown in Figure 6. Pure HDPE displays a typical stress–strain curve of thermoplastics, with yield strength of 28 MPa, Young’s modulus of 1.3 GPa, tensile strength of 32 MPa, and elongation at break of 1,370%. Adding 1.0 wt.% ND leads to a slight increase in yield strength of 31 MPa and a significant improvement in Young’s modulus of approximately 1.8 GPa and approximately 38 % higher than that of pure PE. Though the elongation at break is basically retained compared with pure PE, the presence of ND results in a slight reduction in tensile strength of approximately 28 MPa, which is mostly likely due to its distinct spherical form of ND reducing the intermolecular interaction of PE chains. After incorporating NH_2_-ND into PE via reactive blending, addition of 1 wt.% NH_2_-ND further enhances the yield strength up to 34 MPa relative to HPmND-1%, approximately 21% higher than that of pure PE. Young’s modulus of nanocomposite is also further improved up to 2.0 GPa, approximately 54 % higher than that of pure PE and approximately 11 % higher than that of HPmND-1 %. Like ND, adding of NH_2_-ND still leads to a slight reduction of tensile strength without obvious negative effect on the elongation at break. Recently, Zhang’s studies showed that the incorporation of nano-CaCO_3_ slightly decreased the tensile strength of polylactide, despite of improved elongation at break or toughness [31]; while Mo et al. [32] found that addition of 5 wt.% nano-CaCO_3_ slightly increased the tensile strength to 29.5 MPa relative to neat PP (approximately 27 MPa). However, all mechanical parameters of nanocomposites have little change with further increase in NH_2_-ND loading, which is partially due to the increase in the number of aggregates (see Figure 1D) with increasing loading even if *in situ* reactive compatibilization. Morimune et al.[17] reported that both tensile strength and Young’s modulus increased linearly with increasing ND loading in polyvinyl alcohol (PVA) films, and 1 wt.% ND increased the tensile strength from 95 to 115 MPa, and Young’s modulus was also enhanced from 3.7 to 9.7 GPa. They attributed these remarkable improvements in mechanical properties to strong hydrogen bonding interaction between ND and PVA and high modulus of ND itself. While for our system, the polarity of HDPE is still weak even containing 0.9 wt.% MA groups, and ND is still strongly polar in spite of experiencing a silane modification; and thus, the interfacial compatibility of HDPE and ND is still limited. As for detailed reinforcing mechanism, it is also still under investigation and will be reported along with flame retardancy mechanism in the future.

## Conclusions

HDPE-g-MA/amine-functionalized nanodiamond (NH_2_-ND) nanocomposites were facilely fabricated with homogeneous dispersion of ND particles via one-step reactive melt-blending strategy. The *in situ* reaction of MA groups in HDPE and NH_2_ groups in NH_2_-ND was responsible for the good dispersion of ND. The addition of pristine ND neither has obvious effect nor reduces the thermal stability and thermal oxidation stability of HDPE but NH_2_-ND enhances them due to good dispersion. Both ND and NH_2_-ND delay the time to ignition to different extent and remarkably reduce the heat release rate of HDPE, and the latter performs a little better in terms of enhancing the flame retardancy of polymers. Thus, nanodiamond will be a potential flame retardant for polyolefin or even other polymers. Adding both ND and NH_2_-ND slightly increase the yield strength and significantly enhance the Young’s modulus while without leading to the reduction of elongation at break of polymer host. Whatever, ND seems to display better flame retardancy than nano-CaCO_3_ and exhibit better mechanical reinforcing effects than nano-CB, since few related studies were reported. In addition, the color of nano-CB is also a big problem for some application where light color is required. Thus compared with other cheaper nanoparticles like nano-carbon black or CaCO_3_, ND exhibits comprehensive advantages in terms of improving the flame retardancy and mechanical properties despite its expensive price. Therefore, As-prepared composites will find potential applications such as in building, electric and electronic, and aerospace fields due to their improved flame retardancy and mechanical performances. Both flame retardancy and reinforcing mechanisms are still being investigated in detail and will be reported in the future.

## Competing interests

The authors declare that they have no competing interests.

## Authors’ contributions

SPA and FUY conceived of the experiments and drafted the manuscript. YYM carried out the TEM and IR. WQ and YJW performed the mechanical properties of nanocomposites. All authors read and approved the final manuscript.
